# The Contribution of Hormonal Changes to the Protective Effect of Endophytic Bacterium *Bacillus subtilis* on Two Wheat Genotypes with Contrasting Drought Sensitivities under Osmotic Stress

**DOI:** 10.3390/microorganisms11122955

**Published:** 2023-12-10

**Authors:** Oksana Lastochkina, Ruslan Yuldashev, Azamat Avalbaev, Chulpan Allagulova, Svetlana Veselova

**Affiliations:** Institute of Biochemistry and Genetics—Subdivision of the Ufa Federal Research Centre of the Russian Academy of Sciences, 71 Pr. Oktyabrya, 450054 Ufa, Russiaavalbaev@yahoo.com (A.A.); veselova75@rambler.ru (S.V.)

**Keywords:** plant growth-promoting bacteria (PGPB), endophytic *Bacillus subtilis*, phytohormones, *Triticum aestivum* L., chlorophyll, lignin, lipid peroxidation, electrolyte leakage, tolerance, dehydration

## Abstract

A comparative analysis was conducted to evaluate the effects of seed priming with endophytic bacterium *Bacillus subtilis* 10-4 (BS) on the hormonal system and cell wall tolerance (lipid peroxidation (LPO), electrolyte leakage (EL), and root lignin deposition) of two *Triticum aestivum* L. (wheat) varieties with contrasting drought sensitivities (Ekada 70—drought-tolerant (DT); Salavat Yulaev—drought-sensitive (DS)) under normal conditions and 12% polyethylene glycol-6000 (PEG)-induced osmotic stress. The results showed that under normal conditions, the growth stimulation in wheat plants by BS was attributed to changes in the hormonal balance, particularly an increase in endogenous indole-3-acetic acid (IAA) accumulation. However, under stress, a significant hormonal imbalance was observed in wheat seedlings, characterized by a pronounced accumulation of abscisic acid (ABA) and a decrease in the levels of IAA and cytokinins (CK). These effects were reflected in the inhibition of plant growth. BS exhibited a protective effect on stressed plants, as evidenced by a significantly lower amplitude of stress-induced changes in the hormonal system: maintaining the content of IAA at a level close to the control, reducing stress-induced ABA accumulation, and preventing CK depletion. These effects were further reflected in the normalization of growth parameters in dehydrated seedlings, as well as a decrease in leaf chlorophyll degradation, LPO, and EL, along with an increase in lignin deposition in the basal part of the roots in both genotypes. Overall, the findings demonstrate that BS, producing phytohormones, specifically IAA and ABA, had a more pronounced protective effect on DT plants, as evidenced by a smaller amplitude of stress-induced hormonal changes, higher leaf chlorophyll content, root lignin deposition, and lower cell membrane damage (LPO) and permeability (EL) compared to DS plants.

## 1. Introduction

Drought is a widespread and unpredictable abiotic stress factor that negatively impacts various metabolic pathways in plants, leading to growth inhibition and reduced crop yield, including wheat [1,2,3]. In recent years, the detrimental effects of drought on wheat yield and grain quality have become a pressing issue, exacerbated by climate change [4,5,6]. Wheat is one of the most important cereal crops globally, occupying the largest sowing area and contributing to a substantial grain harvest [4,5]. Wheat grains are processed into flour (for bakery and confectionery products), groats, and other food products, as well as used in animal feed production [5]. Wheat grains are characterized by high protein content, gluten, and excellent baking qualities [6]. However, wheat is also known for its vulnerability to dehydration, resulting in significant yield reduction under water-limited conditions.

The hormonal system plays a crucial role in regulating plant stress resistance and coordinating protective responses during plant adaptation to various stressors [7,8]. In response to water deficiency, the plants typically accumulate abscisic acid (ABA), which is involved in regulating protective responses to dehydration-induced damage [9]. ABA-dependent genes associated with reactive oxygen species (ROS) inactivation and water use efficiency are engaged in transduction and transcription pathways [10,11]. ABA also plays a key role in stress-induced lignin accumulation, enhancing drought resistance in plants [12,13]. Besides ABA, other phytohormones such as indole-3-acetic acid (IAA) and cytokinins (CK) are also involved in regulating plant stress resistance by combining growth-stimulating and protective effects [14,15,16,17]. Each phytohormone has its own mechanisms for modulating genetic programs that control physiological processes during plant development under normal conditions and in response to environmental changes [15,16,17,18,19]. This indicates active cross-interactions among hormones, both in normal and stress conditions [7,8]. Hormones also contribute to the establishment of plant microbiomes, with plants and microbes often employing the same hormones for different purposes [9].

To address the challenges posed by water deficiency and reduce reliance on potentially harmful agrochemicals, environmentally friendly and safe approaches that can activate the natural defense mechanisms of plants against abiotic stresses are being explored. One promising alternative is the use of beneficial plant growth-promoting bacteria (PGPB), particularly species from the *Bacillus* genus [6,20,21,22]. PGPB have shown the ability to enhance plant growth and alleviate the detrimental effects of stresses by inducing natural defense mechanisms [20,23]. Numerous studies have reported the positive effects of PGPB on wheat growth and tolerance under various abiotic stresses [23,24,25,26,27]. The mechanisms underlying PGPB-induced tolerance in wheat involve the modulation of phytohormones, regulation of photosynthesis and water metabolism, synthesis of osmoprotective and antioxidant compounds, as well as other metabolites, and enhancement of the bioavailability of essential macro- and micronutrients (recently reviewed in detail [21,22,28,29]). Current knowledge indicates that liquid culture filtrates of *Bacillus* spp. may contain various phytohormones including ABA, IAA, and CK [30,31], which play crucial roles in photosynthesis, plant growth, plasma membrane integrity, and microbe-induced systemic tolerance [28,29]. *Bacillus* spp. utilize the IAA they produce as part of their colonization strategy when interacting with plants. This includes stimulating plant growth and bypassing the plants’ main defense mechanisms. Studies have demonstrated that IAA-producing *Bacillus* bacteria significantly enhance the growth of wheat plants under normal conditions and drought stress [32,33] by increasing water and nutrient uptake. The ability to produce IAA is most commonly found among soil bacteria and is more prevalent among endophytic bacteria than epiphytic bacteria [34,35]. Approximately 90% of P-solubilizing bacteria have been found to possess the ability to produce CK in vitro [36]. PGPB strains that produce ABA have been shown to increase internal ABA levels and enhance drought tolerance in plants [37] by modulating other endogenous hormones, upregulating essential amino acids (including osmoregulators) [38], reducing leaf transpiration, regulating stomatal conductance, and inducing the expression of genes involved in drought tolerance mechanisms [28,29]. Despite these fundings, the mechanisms by which PGPB affect endogenous hormone levels and enhance plant stress tolerance are not fully understood yet [39]. However, many aspects of the interaction between PGPB and wheat plants under dehydration remain unclear and require further investigation to fully harness their potential. The effectiveness of PGPB-induced plant tolerance formation is largely determined by the concentration changes in phytohormones relative to each other, as well as the ability of each hormone to affect the endogenous level of others [40]. Additionally, recent findings suggest that effectiveness of the same microbial strain can vary depending on plant genotype, stress type, and other factors [41,42]. Therefore, it is crucial to explore the role of the hormonal system in the PGPB-mediated regulation of molecular mechanisms in wheat, a key crop, under dehydration stress. Despite accumulated data on the ability of PGPB to reduce drought damage, their specific influence on the plant hormonal system and their contribution to bacterial-mediated drought tolerance in different wheat genotypes with varying sensitivity to drought remain unclear. Further research is needed to address these gaps in knowledge and fully understand the potential of PGPB in improving drought tolerance in wheat. We hypnotized that endophytic bacterium *B. subtilis* 10-4 (BS), known for its plant growth-promoting properties [40,41,42,43], enhances tolerance of wheat genotypes contrasting in drought sensitivity differently influencing the hormonal balance, specifically ABA, IAA, and CK, which are involved in protective responses and stress adaptation in plants.

This work is devoted to the study of the effects of endophytic bacterium *B. subtilis* 10-4 on the hormonal status of two wheat genotypes with contrasting drought sensitivities exposed to 12% polyethylene glycol-6000 (PEG) which caused osmotic stress, as well as assessment of plant growth and cell wall tolerance parameters.

## 2. Materials and Methods

### 2.1. Bacterial Strain and Inoculum Preparation

The endophytic bacterium BS was earlier isolated from the arable soils of the Republic of Bashkortostan (52°36′ N 58°19′ E, Russia), identified using 16s rRNA analysis, described in detail in our previous works [42,43] and deposited in the National Bioresource Center of the All-Russian Collection of Industrial Microorganisms NRC “Kurchatov Institute” (No. B-12988, dated 23 June 2019). Based on the results of the sequencing analysis of the variable regions of genes encoding 16S rRNA as well as PCR analysis using species-specific primers, strain 10-4 was identified as *B. subtilis* (99%) [42]. Using surface-sterilized wheat seedlings, *Bacillus* ChromoSelect Agar and Random amplification of polymorphic DNA—polymerase chain reaction (RAPD–PCR) analysis demonstrated the ability of BS to colonize inner wheat tissues (endophytic existence) [40]. Strain 10-4 is capable of producing IAA, siderophores, and fix atmospheric N2 [32]. The cells of BS were cultured in a liquid Luria–Bertani (LB) medium for 24 h (37 °C, 180 rpm) [44]. To produce inoculum, freshly obtained liquid bacterial culture containing 10^9^ cells mL^−1^ was diluted down to 10^5^ cells mL^−1^ using sterile water. The concentration was selected previously as optimal in wheat growth promotion and protection under stress conditions [42]. The concentration of bacterial inoculum was monitored by the optical density at 600 nm (SmartSpecTM Plus spectrophotometer, Bio-Rad, Hercules, CA, USA).

### 2.2. Plant Materials, Experimental Design, and Growth Conditions

The experiments were carried out in hydroponically grown two soft spring wheat (*Triticum aestivum* L.) genotypes contrasting in drought sensitivity (i.e., drought-tolerant (DT) Ekada 70 variety and drought-susceptible (DS) Salavat Yulaev variety) [40]. Wheat seeds were obtained from the Chishminsky Breeding Station of the Ufa Federal Research Centre of Russian Academy of Sciences (UFRC RAS) (Chishmy, Ufa, Russia). The seeds were sterilized in 96% ethanol (C_2_H_5_OH) for 1 min, then washed 4–5 times with sterile water. Before sowing, the sterilized seeds were inoculated with BS by immersion into the suspension of bacteria (10^5^ cells mL^−1^) or water (control) for 1 h. Then, the seeds were grown on filter paper moistened with sterile water under a long-day photoperiod (16 h light/8 h dark, 200 μmoL m^−2^ s^−1^, 21–24 °C). Seedlings aged 4 days were transferred into glasses with water (control) or 12% polyethylene glycol-6000 (PEG) (stress), and after different time periods they were fixed for estimation of phytohormones (1, 3, 7 h), lignin (24 h). The growth parameters and chlorophyll (Chl) content were analyzed after 72 h of stress exposure.

The length of roots and shoots as well as their fresh and dry biomass were assessed according to [45]. Leaf Chl content (Chl a and Chl b) was assayed as described [46]. In brief, seedling leaves (0.05 g) were extracted in 90% C_2_H_5_OH (10 mL) with the addition of calcium carbonate (CaCO_3_). Pigment absorption was measured at 663 nm (Chl a) and 646 nm (Chl b) using a SmartSpecTM Plus spectrophotometer (Bio-Rad, Hercules, CA, USA).

### 2.3. Phytohormone Extraction and Quantification in Wheat Seedlings and in the Liquid Culture Medium of Bacteria

The content of endogenous phytohormones was determined in 4-day-old seedlings exposed to 12% PEG-6000 for 1, 3, and 7 h. The content of free IAA, ABA, and CK in one plant sample was determined by the enzyme-linked immunosorbent assay (ELISA) using rabbit antibodies specific to each of these hormones and anti-rabbit antibodies labeled with peroxidase as described in [47,48]. To achieve this, a sample of 10 seedlings was triturated in liquid nitrogen (N2) and phytohormones were extracted with 80% C_2_H_5_OH for 16 h at 4 °C. After centrifugation for 10 min at 10,000× *g*, the supernatant was evaporated in a stream of air to an aqueous residue, in an aliquot of which the total content of zeatin derivatives (zeatin, its riboside and nucleotide) highly immunoreactive to the serum obtained to zeatin riboside was determined [48,49,50]. Immunoassay reliability was confirmed by dilution tests, chromatographic examination of the distribution of immunoreactivity, and comparison of the results of immunoassay against physicochemical assays (liquid chromatography–mass spectrometry) [51,52]. For IAA and ABA estimation, the remaining aqueous residue was acidified with hydrochloric acid (HCl) to pH 2.5 and partitioned twice with diethyl ether (C_2_H_5_)_2_O). Then, IAA and ABA were transferred from the organic phase into 1% sodium hydrocarbonate (NaHCO_3_) (pH 7–8), re-extracted with (C_2_H_5_)_2_O, methylated with diazomethane (CH_2_N_2_) and immunoassayed using antibodies to ABA and IAA [48,53]. The reliability of the immunoassay for IAA and ABA was ensured by both the high specificity of antibodies and effective prior purification of hormones according to a modified scheme of solvent partitioning [53].

The ability of BS to produce phytohormones (IAA, ABA, and CK) was tested on an LB medium. The determination was performed using the enzyme-linked immunosorbent assay (ELISA) method with specific rabbit and secondary anti-rabbit antibodies (Sigma-Aldrich, Saint Louis, MO, USA) [47]. BS cells were cultured in an LB medium at 180 rpm, 37 °C for 24 h. The cells were then pelleted by centrifugation at 5000 rpm for 10 min. In a 5 mL aliquot of the bacterial culture medium, the total content of immunoreactive CK forms was determined. The remaining aqueous residue was extracted with (C_2_H_5_)_2_O for IAA and ABA hormones. IAA and ABA hormones were methylated with CH_2_N_2_, and after evaporation, the dry residue was dissolved in 80% C_2_H_5_OH. The amount of ABA was determined in an aliquot of the dissolved residue. The purification and extraction procedure for IAA and ABA, as well as the sequence of steps for immunoanalysis of phytohormones, are described above. Each analysis was carried out in two independent experiments, each of which was performed in three biological and five analytical replicates.

### 2.4. Assessment of Lignin Deposition in Roots

The lignin deposition in the basal part of roots was determined using phloroglucinol staining according to [54] with modifications. To achieve this, the longitudinal sections were made from the basal part of the roots of living plants using a LeicaCM1520 cryostat (Leica Biosystems, Nussloch, Germany) at −30 °C. For pouring, a sample freezing liquid (Leica Biosystems, Tissue Freezing Medium, Nussloch, Germany) was used; the slice thickness was 60 μm. Then, the cut root sections were immediately placed on a glass slide in a 5% C_2_H_5_OH solution of phloroglucinol (C_6_H_6_O_3_) for 3 min. Slides were treated with 3–4 drops of 10% HCl and imaged using a fluorescence scanning microscope (Biozero BZ–8100E, Keyence Co., Osaka, Japan). The degree of lignin deposition was assessed on a scale of staining intensity [55].

### 2.5. Measurement of Lipid Peroxidation (LPO) and Electrolyte Leakage (EL)

The intensity of LPO was judged by the content of malondialdehyde (MDA) in seedlings using a color reaction with thiobarbituric acid [56]. The optical densities of tested samples were measured using a SmartSpecTM Plus spectrophotometer (Bio-Rad, Hercules, CA, USA) at 532 nm.

The permeability of the cell membranes of seedlings was judged from the electrolyte leakage (EL), which was recorded using an OK 102/1 conductometer (Radelkis, Budapest, Hungary), measuring the ohmic resistance of aqueous extracts in direct current [57].

### 2.6. Statistical Analysis

All experiments were carried out in three biological and three–five analytical replicates. Data were processed statistically by the analysis of variance (ANOVA) technique using program STATISTICA 6.0. Significant differences between treatments were compared by using the least significant difference (LSD) test (*p* < 0.05). The figures and tables show the means and their standard errors (±SE). The calculation of Pearson’s correlation coefficients and the construction of correlation matrices were carried out using the Data Analysis ToolPak in Excel 2016.

## 3. Results

### 3.1. The Ability of Strain Bacillus subtilis 10-4 (BS) to Produce Phytohormones Indole-3-Acetic Acid (IAA), Cytokinins (CK), and Abscisic Acid (ABA)

**Figure 1:**
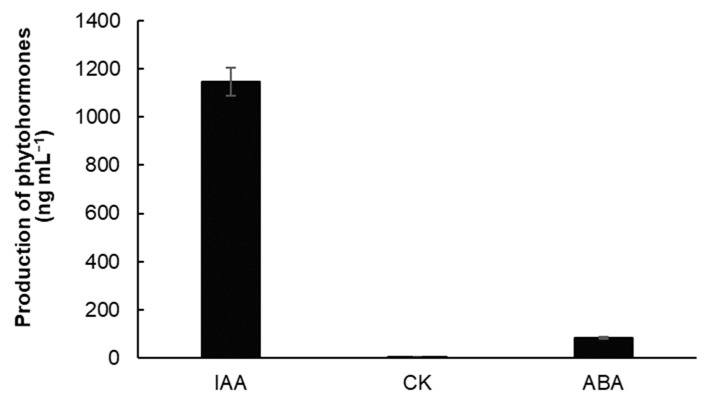
The content of phytohormones indole-3-acetic acid (IAA), cytokinins (CK), and abscisic acid (ABA) in the culture medium of strain *Bacillus subtilis* 10-4 (BS) after a 24 h incubation in liquid Luria–Bertani (LB) medium (37 °C, 180 rpm). The data were obtained in two independent experiments, each of which was performed in three biological and five analytical replicates. Error bars represent standard errors (±SE) of the means.

The analysis of the LB culture medium of BS after 24 h of cultivation showed the presence of the following phytohormones: IAA (1147 ± 55 ng mL^−1^), ABA (85 ± 4.1 ng mL^−1^), as well as trace amounts of CK (3 ± 0.1 ng mL^−1^) (Figure 1).

### 3.2. The Growth Attributes (Length, Biomass, Leaf Chlorophyll) of Endophyte-Primed Hydroponically Grown Wheat Seedlings under Normal and Stress Conditions

The influence of dehydration modeling by PEG on shoot and root length, shoot and root fresh (FW) and dry (DW) weight and chlorophyll content (chlorophyll a + chlorophyll b) of non-bacterial (−BS) and bacterial-primed (+BS) plants was studied.

It was found that non-primed plants of both varieties demonstrated a notable decrease in growth attributes under osmotic stress (Table 1). However, DT and DS plants differed in their sensitivity to stress. The results showed that dehydration differently decreased shoot FW (by 23% and 31%), root DW (by 20% and 44%), root FW (by about 5% and 30%), root DW (by 5% and 38%), and chlorophyll content (by 11% and 30%) of DT and DS plants, respectively. These data clearly demonstrate the contrasting drought resistance of the two varieties.

Seed priming with BS had a positive influence on all tested growth attributes of DT plants and only some parameters of DS plants under normal conditions (Table 1). BS increased the shoot and root lengths (by 14% and 29%), shoot and root FW (by 15% and 20%) and DW (by 14% and 9%) for the DT genotype, while for the DS genotype, there were increases in shoot FW and DW (by 16% and 13%) and root FW and DW (by 14% and 17%) and insignificant changes in shoot and root lengths. Additionally, under normal conditions, BS increased (by 12%) leaf Chl (Chl a + Chl b) in DT plants, while there was no significant difference observed in DS plants compared to the non-primed control groups.

Under dehydration conditions, BS-primed seedlings of both varieties exhibited improved growth attributes compared to untreated plants (Table 1). However, in BS-primed and stressed DT plants, these parameters were significantly increased and even exceeded the level of control non-stressed control plants. Specifically, there was a 7% increase in root length, a 13% increase in root DW, and a 6% increase in Chl content.

Thus, the results indicate that the response to BS priming in wheat seedlings is specific to variety. Better growth attributes were observed in DT wheat seedlings upon BS priming, under non-stress and stress conditions.

### 3.3. Endogenous Phytohormones Abscisic Acid (ABA), Indole-3-Acetic Acid (IAA), and Cytokinins (CK) Concentrations in Endophyte-Primed Seedlings under Normal and Osmotic Stress Conditions

**Figure 2:**
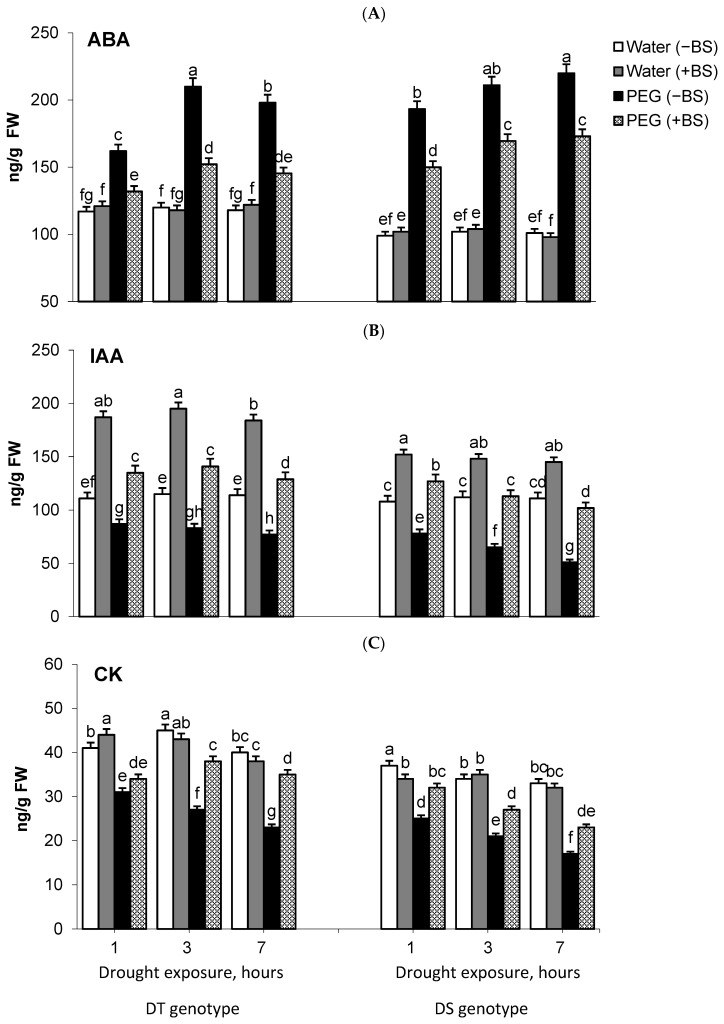
Effect of seed priming with endophytic *B. subtilis* 10-4 (BS) on the changes in plant hormonal balance, i.e., the content of endogenous abscisic acid (ABA) (**A**), indole-3-acetic acid (IAA) (**B**), and cytokinins (CK) (**C**) in drought-tolerant (DT) and drought-sensitive (DS) wheat seedlings grown under non-stress conditions (water) and 12% polyethylene glycol-6000 (PEG) conditions, which caused osmotic stress. The stress exposure durations were 1, 3 and 7 h (i.e., in 4 days after planting and growing under normal conditions, the seedlings were transferred into a PEG solution for 1, 3, and 7 h to induce stress). Values are presented as mean±SE (*n* = 3). Different letters on top of the columns indicate that means for different treatments for one genotype indicate a statistically significant difference at *p* < 0.05 (ANOVA, LSD test).

Endogenous phytohormones ABA, IAA, and CK were quantified in both BS-primed and non-primed plants with contrasting drought sensitivities under normal and osmotic stress conditions (Figure 2). It was found that the control plants of the DT variety differed from those of the DS variety by a higher content of CK and ABA, while the concentrations of IAA were similar in both varieties (Figure 2A–C).

Dehydration resulted in significant changes in the hormonal balance of plants in both varieties, characterized by an increase in ABA accumulation (Figure 2A) and a gradual decrease in the levels of IAA (Figure 2B) and CK (Figure 2C). However, more dramatic changes in hormone concentrations were observed in the DS genotype, which corresponded to stronger growth inhibition (Table 1).

Priming with BS demonstrated a significant protective effect on plants of both varieties under drought conditions. This effect was particularly evident in the DT variety, which showed significantly lower amplitude of stress-induced changes in the hormonal system (Figure 2) and higher growth rates in wheat plants during prolonged stress conditions (Table 1).

### 3.4. Lignin Accumalation in Roots of Endophyte-Primed Seedlings under Normal and Osmotic Stress Conditions

**Figure 3:**
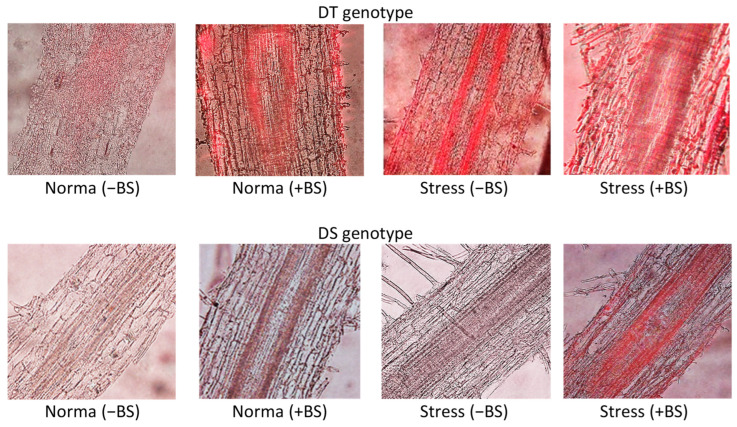
Effect of seed priming with endophyte *B. subtilis* 10-4 (BS) on lignin deposition in basal part of roots of drought-tolerant (DT) and drought-sensitive (DS) wheat seedlings grown under non-stress (water) and osmotic stress conditions, caused by 12% polyethylene glycol-6000 (PEG) (*n* = 3). Time of stress exposure—24 h (i.e., in 4 days after planting, the seedlings grown under normal conditions were transferred into a PEG solution for 24 h to induce stress). Scale bars = 100 µm.

A comparative analysis of lignin deposition was conducted in the longitudinal sections of the basal roots of endophytic BS-primed and unprimed seedlings under normal conditions and under the presence of PEG (Figure 3). The results revealed a difference in lignin deposition between the DT and DS plants under normal conditions. The DT variety exhibited significant lignin accumulation, whereas the DS variety showed very weak lignin deposition (Figure 3, Table 2).

After incubating wheat seedlings in PEG stress for 24 h, there was an increase in lignin deposition in the roots, predominantly in the cell walls of the central cylinder and to a lesser extent in pericycle and primary cortex cells. The intensity of lignin deposition was higher in the DT genotype compared to the DS genotype, particularly in the central cylinder and primary cortex cells (Figure 3, Table 2).

Under normal growth conditions, seed priming with the endophyte (+BS) resulted in an accelerated deposition of lignin deposition in the cell walls of the central cylinder, pericycle, and primary cortex in the roots of both genotypes compared to the control. However, the DT genotype exhibited higher levels of lignin deposition compared to the DS genotype (Figure 3, Table 2). Furthermore, in BS-primed and stressed wheat seedlings of both genotypes, there was an additional intensification of lignin deposition in the cell walls of the central cylinder, pericycle, and primary cortex cells. Notably, the highest levels of lignin content were observed in the BS-primed DT genotype compared to the BS-primed DS genotype under the same stress conditions (Figure 3, Table 2).

### 3.5. Lipid Peroxidation (LPO) and Elektrolyte Leakage (EL) Degree in Endophyte-Primed Seedlings under Osmotic Stress

**Figure 4:**
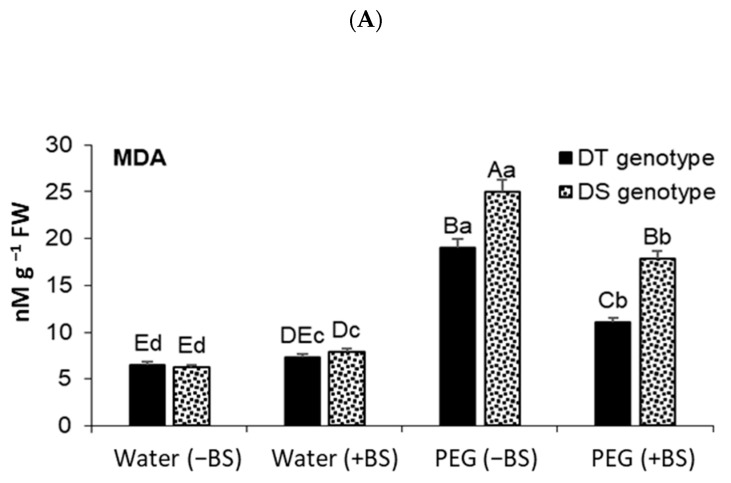

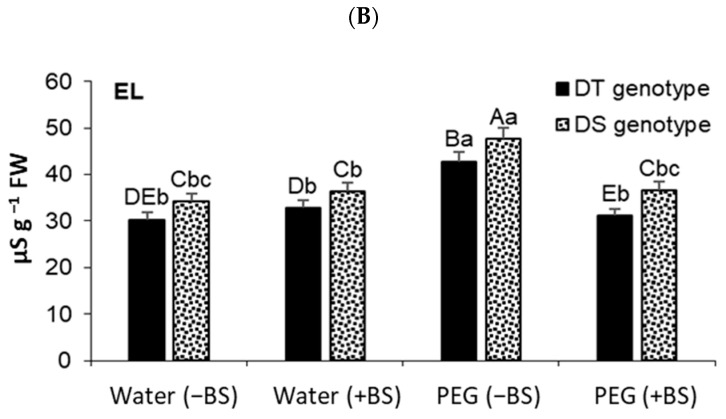
Effect of endophyte *B. subtilis* 10-4 (BS) priming on lipid peroxidation (malondialdehyde (MDA)) (**A**) and electrolyte leakage (EL) (**B**) of drought-tolerant (DT) and drought-sensitive (DS) wheat seedlings grown under osmotic stress (12% polyethylene glycol-6000 (PEG)) conditions for 24 h (i.e., in 4 days after planting, the seedlings grown under normal conditions were transferred into a PEG solution for 24 h to induce stress). Values are presented as mean ± SE (*n* = 3). Different lowercase letters are indicating a statistically significant difference between treatments for one genotype at *p* < 0.05 (ANOVA, LSD test). Different capital letters are indicating a statistically significant difference between two genotypes at *p* < 0.05 (ANOVA, LSD test).

The studied wheat varieties initially showed slight differences in the level of MDA (Figure 4A) and EL (Figure 4B) under normal conditions. However, incubating the seedlings in a medium containing PEG resulted in increased levels of these indicators in both varieties (Figure 4A,B). The increase in EL was comparable between the two varieties (Figure 4B), while the DS variety exhibited a nearly one-third increase in MDA content compared to the DT variety (Figure 4A).

Priming with BS had a significant impact on reducing the levels of MDA and EL in wheat varieties with different drought tolerance (Figure 4A,B). This indicates a positive effect of the bacteria on maintaining the integrity of cell membranes during dehydration.

In summary, the results indicate that the protective effect of endophytic BS on wheat plants during drought is associated with the restructuring of hormonal balance. This includes maintaining a higher level of IAA, CK while preventing stress-induced increase in ABA. Furthermore, bacterial treatment had a more pronounced protective effect on the DT variety, as evidenced by a smaller amplitude of stress-induced restructuring of the hormonal system, normalization of growth, and improved cell wall tolerance parameters under stress conditions.

### 3.6. Correlation Matrices

**Figure 5:**
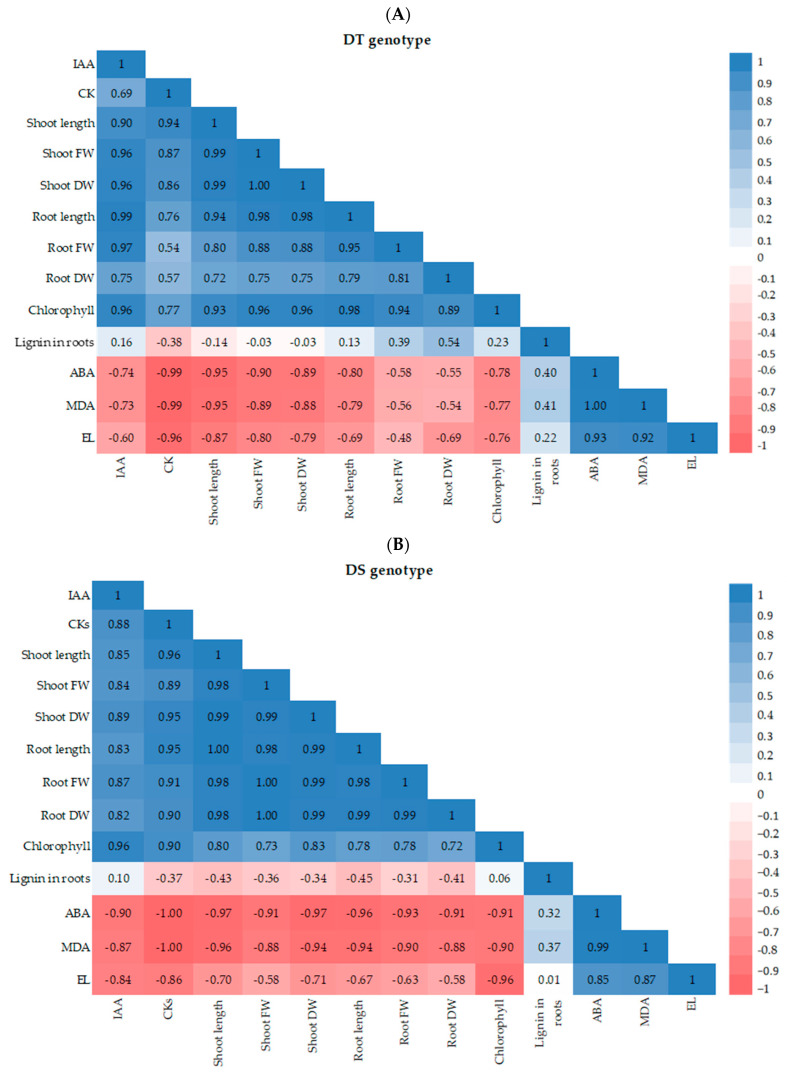
The correlation coefficient matrix for all parameters examined in this study for drought-tolerant (DT) genotype Ekada70 (**A**) and drought-sensitive (DS) genotype Salavat Yulaev (**B**) colored by magnitude and parity (blue positive and red negative) for correlations at *p* < 0.05 significance level.

Factor analysis revealed that all the studied indicators of both wheat genotypes could be grouped into two clusters, with positive correlations within each cluster and negative correlations between the clusters (Figure 5. The first cluster included endogenous IAA and CK, growth indicators (shoot and root length, FW, and DW), and total Chl content. The second cluster included endogenous ABA, lignin in the roots, and markers of oxidative stress (MDA and EL). These findings support the understanding that auxins and CK are hormones associated with plant growth and development, while ABA is linked to stress responses. Lignin content showed the least correlation with other indicators in the second cluster.

Regarding the difference between wheat genotypes, two notable points stand out. First, in the DT genotype, growth indicators and Chl content are more strongly correlated with the accumulation of endogenous IAA. In contrast, in the DS genotype, there is a slightly stronger correlation between these parameters and the accumulation of CK. This suggests that IAA plays a more prominent role in driving growth in the DT genotype, while CK may have a greater impact in the DS genotype. The second difference pertains to the correlation between lignin deposition and root growth indicators. In the DT genotype, lignin deposition does not significantly affect root growth indicators. However, in the DS genotype, lignin deposition is associated with the inhibition of root growth.

## 4. Discussion

Drought is a significant challenge that limits crop production. Extensive research is being conducted to understand how wheat genotypes respond to dehydration under the influence of PGPB [21,22]. Comparative studies involving wheat varieties with contrasting drought susceptibility play a crucial role in unraveling the mechanisms underlying plant adaptation to water deficit [41,58,59,60,61,62]. Our results showed that the DT wheat variety is more resistant to drought than DS variety: drought decreased the length of seedlings, biomass, and leaf Chl content of DT variety significantly less than DS variety (Table 1). The ability of PGPB to mitigate drought-caused damages in various plants, including wheat, are well documented [21,23,28,29,63,64,65]. However, the knowledge about the responses of drought-tolerant and susceptible wheat genotypes upon endophytic PGPB treatment is still limited. Our results showed that bacterial priming significantly improved the growth parameters of DT variety more than they did those of the DS variety (Table 1), as well as increased cell wall tolerance under drought conditions, as evidenced by reduced LPO, EL (Figure 4), and accelerated lignification of root cell walls (Figure 3, Table 2).

Under conditions of water deficit, plants experience oxidative stress resulting from excessive generation of ROS, which can cause damage to membrane structures [3]. Avoiding damage to cell membranes is a crucial factor for plants to withstand drought stress. Certain PGPBs have shown the ability to assist plants in this aspect. Studies have demonstrated that wheat plants inoculated with bacteria exhibit reduced production of ROS and increased activity of antioxidant enzymes under stress [24,25,41,66,67]. Additionally, research has indicated that wheat seedlings inoculated with PGPB *Klebsiella* sp. IG 3 exhibit lower EL and MDA levels compared to non-inoculated plants under drought conditions [68]. EL and MDA are reliable indicators of oxidative membrane damage caused by stress. Our results also revealed that both DT and DS seedlings pretreated with BS and subjected to PEG-induced dehydration exhibited a decrease in the stress-induced MDA accumulation (Figure 4A) and EL (Figure 4B). Notably, the bacterial treatment had a more pronounced protective effect on DT plants, as evidenced by reduced cell membrane damage (LPO) and permeability (EL) under stress.

Lignin deposition is a crucial mechanism for enhancing the drought resistance of plants [14,69,70]. A well-known player in the stress-induced accumulation of lignin in plants is hormone ABA [13,14]. Additionally, it has been reported that bacteria can enhance lignin accumulation, thereby contributing to the formation of apoplastic barriers [71,72]. Given that BS treatment increased the drought resistance of both wheat varieties while also increasing endogenous ABA content, we further investigated the effect of bacterial treatment on lignin deposition in the roots of wheat plants. It is worth noting that at the start of our work, we had no information regarding the effect of endophytic PGPBs on lignin deposition in wheat plants with contrasting drought sensitivity under osmotic stress. Our findings revealed that lignin deposition contributes to the endophyte-induced drought resistance of wheat plants (Figure 3, Table 2). This conclusion was based on the observation of higher lignin accumulation in bacterial-inoculated seedlings of both genotypes under normal and particularly stress conditions. Moreover, this protective mechanism was more prominent in enhancing dehydration resistance in the DT variety. The results highlight the significant role of endogenous ABA in regulating the bacterial-induced acceleration of lignin deposition acceleration in the cell walls of wheat roots, which can contribute to strengthening their barrier properties. Importantly, the observed lignin accumulation induced by BS (Figure 3, Table 2) did not hinder the growth-promoting effect of the bacteria (Table 1), likely due to the bacteria’s ability to produce IAA (Figure 1) and modulate endogenous IAA levels in plants (Figure 2B), as well as produce other metabolites with growth-promoting properties [43]. According to factor analysis (Figure 5), IAA and CK are hormones associated with plant growth and development, while ABA is linked to stress responses. Additionally, lignin content showed the least correlation with MDA and EL. This could be attributed to the fact that lignin deposition is not exclusively stress induced, as seed priming with the bacterium also led to increased lignin deposition in the roots (Figure 3 and Figure 5, Table 2). Moreover, the results indicate that the impact of lignin on root growth is more pronounced in the DS genotype than in the DT genotype (Figure 5).

The hormonal system plays a key role in both plant development (ontogenesis) and the plant’s ability to adapt to environmental stresses [8,73]. The adaptation to stress is closely linked to the balance of endogenous phytohormones [74,75]. When plants experience stress, such as drought, typical responses include alterations in hormonal balance characterized by a temporary increase in stress hormone ABA and a decrease in the levels of IAA and CK [75,76]. In our study, the analysis of endogenous ABA, IAA, and CK in DT and DS plants under normal conditions revealed that DT plants exhibited higher concentrations of CK and ABA compared to the DS variety, while the levels of IAA were maintained at similar levels in both varieties (Figure 2A–C).

ABA is a crucial phytohormone that plays a central role in regulating response to stress [7,77,78,79,80,81]. Its protective role is essential for plant growth, as it helps to close stomata in leaves to reduce water loss and activates various genes related to dehydration stress, such as those involved in minimizing transpiration or combating oxidative stress [82,83,84,85]. Consequently, ABA protects plants from further water loss and damage while enhancing plant stress tolerance [79,86,87]. Significant genotypic variations in ABA accumulation in wheat leaves under water stress have been observed in numerous studies conducted on wheat (reviewed by Saradadevi et al. [81]). Wheat genotypes that accumulate less ABA have been associated with drought tolerance, while those that accumulate higher levels of ABA are considered sensitive to drought [88,89]. According to these data, in our study, water stress resulted in a significant increase in ABA accumulation in the seedlings of DS wheat compared to the DT variety (Figure 2A). Additionally, there was a persistent decrease in the levels of IAA and CK (Figure 2B,C), which led to the inhibition of growth processes in these plants (Table 1).

Plant–microbial interactions have been reported to mitigate the adverse effects of abiotic stress by reducing ABA levels [87,90,91,92,93]. For example, wheat seedling primed with the *B. subtilis* strain LDR2 showed a lower concentration of ABA during drought stress compared to non-inoculated seedlings [33]. Similar results were observed in our study, where inoculation with BS led to a decrease in ABA levels (Figure 2A) and an increase in wheat plant growth under stress (Table 1). It should be noted that various bacteria have been reported to increase ABA levels in plants as well [78,94]. Bacterial-mediated changes in ABA levels may impact stomatal conductance, which plays a role in maintaining photosynthesis [78]. This, in turn, can affect wheat yield [22,32,33,95]. In wheat, the amount of ABA is often negatively correlated with photosynthesis efficiency and leaf Chl content [86]. The observed increase in Chl concentration in wheat plants treated with *P. plecoglossicida* 2,4-D was found to be correlated with a decrease in ABA concentration in plant shoots during water deficit [96]. Similar results were obtained in our study, where BS-treated wheat plants showed comparable outcomes under osmotic stress (Table 1). The increased accumulation of Chl in PGPB-treated and stressed plants is likely attributed not only to the hormonal effect on Chl metabolism but also to an increase in root biomass. This improvement in root biomass enhances the roots’ ability to absorb nitrogen, which is essential for Chl synthesis [22,96]. So, plant colonization with PGPB can activate multiple mechanisms that assist plants in coping with stress conditions, including maintaining photosynthesis [22]. Furthermore, the mechanisms of bacterial-induced ABA regulation in plants can vary depending on the characteristics of the specific strain and plant genotype.

Auxins play a crucial role in regulating plant growth and development. They can increase the mitotic activity of plant tissues and stimulate the activity of enzymes involved in cell wall strength, thereby promoting cell elongation and overall growth [8,97]. The most common naturally occurring plant hormone of the auxin class IAA (indole-3-acetic acid) is a potent signaling molecule that plays a vital role in plant–microbial interactions and directly enhances plant growth. It increases the pool of auxins in plants, leading to cell elongation, vascular tissue development, and apical dominance [98]. Under normal growth conditions, BS treatment resulted in a nearly 2-fold increase in IAA levels (Figure 2B), while the content of ABA and CK remained relatively stable (Figure 2A,C). Additionally, DT plants exhibited significantly higher accumulation of IAA. This could explain the positive influence of bacterial treatment on various growth attributes of DT plants and some parameters of DS plants under normal conditions (Table 1).

There is a lot of information that IAA-producing microorganisms play a significant role in enhancing plant growth under stress conditions. They participate in the regulation of stress-associated genes [99], initiate auxin-sensitive gene signaling in plants [100], activate antioxidant enzymes, promote the synthesis of osmoprotectors, and enhance the accumulation of photosynthetic pigments [41,94,97]. These mechanisms collectively contribute to improved plant resilience and performance under stress. Wheat seedlings primed with *B. subtilis* LDR2 exhibited an increase in IAA content and improved photosynthetic efficiency under drought stress compared to non-inoculated plants. This effect is attributed to bacterial capacity to enhance plant IAA levels by modulating the synthesis and signaling pathway of IAA [94]. PGPBs that produce IAA play a role in mediating primary root elongation and promoting lateral root development [101,102,103]. These bacteria have also been shown to enhance wheat yield under drought stress conditions [103]. The altered root architecture resulting from the actions of IAA-producing bacteria leads to improved water absorption by plants and enhanced availability of minerals for their nutrition [104,105]. In line with these findings, our study demonstrated that seed priming with the endophytic bacterium BS had a growth-enhancing effect and significantly alleviated the negative impacts of drought on wheat plants (Table 1). This positive effect can be attributed, in part, to the production of IAA by BS, as demonstrated in both our previous study [42] and current research (Figure 1).

CK are involved in the development of protective responses against biotic and abiotic stress factors [106,107]. Our results suggest the potential involvement of CK in drought stress tolerance, as indicated by the higher basal level of CK in the DT genotype (Figure 2C). However, it appears that endogenous CK is not directly involved in the physiological actions of the endophytic bacterium. This is supported by insignificant changes in CK levels observed in both bacterial-treated wheat genotypes under normal conditions, as well as a decrease in CK levels compared to the enhanced IAA content (even higher than the control) under stress conditions (Figure 2B,C). This can be attributed to the fact that BS primarily produces auxins, not CK (Figure 1). Several studies have demonstrated that CK-producing PGPB can increase endogenous CK levels in plants, thereby contributing to enhanced plant stress resistance [108,109]. The findings of our study suggest that the ability of BS to produce phytohormones, specifically IAA and ABA, may play a significant role in its growth-promoting and stress-mitigating effects, respectively, particularly by modulation endogenous hormonal changes. There is ample literature supporting the ability of endophytic PGPB to synthesize IAA, with around 34% of endophytes possessing this capability [110]. However, information regarding CK-producing bacteria, especially in the context of endophytes, is relatively limited and primarily associated with rhizosphere microorganisms [111]. Our results demonstrate that endophytic BS produce a substantial amount of IAA but minimal ABA and traces of CK (Figure 1). Therefore, BS exhibited a growth-promoting effect on DT wheat plants and alleviated drought-induced damage, partly due to the production of phytohormones IAA and ABA.

In general, seed priming with endophytic bacterium BS resulted in a significant reduction in stress-induced fluctuations in the balance of phytohormones. This was seen as a decrease in the accumulation of endogenous ABA and the prevention of a decrease in IAA levels, as well as partial decrease in stress-induced CK levels (Figure 2). Furthermore, in plants that were pretreated with the endophyte and subjected to stress, the content of IAA remained higher compared to the control throughout the experiment, albeit lower than the level of IAA in the samples pretreated with the endophyte under normal conditions (Figure 2B). This is likely the reason for the best growth attributes observed in the DT genotype under stress conditions (Table 1). Thus, comparative analysis of phytohormone balance in pretreated and untreated seedlings revealed a more positive effect of bacteria on the hormonal status of DT wheat seedlings during PEG-induced osmotic stress.

## 5. Conclusions

The data obtained from the study indicates that seed priming with endophytic bacterium BS producing IAA and ABA combines growth promotion and induction of drought tolerance in wheat seedlings, with the most significant effects observed in the DT genotype. An important factor contributing to the protective action of BS is its ability to influence the endogenous hormonal system of seedlings. The study revealed that the anti-stress effect induced by BS in wheat plants under drought conditions is achieved through the modulation of hormonal balance, including maintaining elevated levels of IAA and CK while reducing stress-induced ABA accumulation. Furthermore, in the DT genotype, seed priming with BS and subsequent dehydration resulted in significantly lower amplitude of stress-induced changes in ABA and IAA levels, along with the maintenance of higher CK levels. Another ABA-dependent mechanism underlying the protective effect of BS on wheat plants under dehydration is its ability to enhance plant cell wall tolerance. The study demonstrated that in the DT genotype, BS priming had a more positive impact on stress tolerance indicators such as MDA and EL, as well as lignin deposition in roots under both normal and stress conditions.

## Figures and Tables

**Table 1:** Effect of *B. subtilis* 10-4 (BS) seed priming on root and shoot length, fresh (FW) and dry (DW) biomass, and leaf chlorophyll (Chl) (Chl a + Chl b) of wheat seedlings grown under non-stress conditions (water) and 12% polyethylene glycol-6000 (PEG) conditions, which caused osmotic stress (*n* = 3). Time of stress exposure was 72 h (i.e., in 4 days after planting, the seedlings grown under normal conditions were transferred into PEG solution for 72 h). Different letters in one measure indicate a statistically significant difference at *p* < 0.05 (ANOVA, LSD test).

Treatments		Shoot Length (cm)	Root Length (cm)	Shoot (g)		Root (g)		Chl Content (mg g^−1^ FW)
				FW	DW	FW	DW	
		DT genotype						
Water	(−BS)	6.9 ± 0.2 ^b^	5.8 ± 0.1 ^b^	0.51 ± 0.02 ^b^	0.05 ± 0.008 ^b^	0.55 ± 0.01 ^c^	0.055 ± 0.009 ^b^	1.23 ± 0.08 ^c^
	(+BS)	7.9 ± 0.3 ^a^	7.5 ± 0.2 ^a^	0.59 ± 0.03 ^a^	0.06 ± 0.006 ^a^	0.66 ± 0.04 ^a^	0.060 ± 0.003 ^ab^	1.38 ± 0.11 ^a^
PEG	(−BS)	4.1 ± 0.1 ^d^	4.5 ± 0.2 ^c^	0.39 ± 0.04 ^c^	0.04 ± 0.002 ^c^	0.53 ± 0.01 ^d^	0.052 ± 0.006 ^c^	1.10 ± 0.06 ^d^
	(+BS)	6.6 ± 0.2 ^c^	6.2 ± 0.3 ^b^	0.51 ± 0.01 ^b^	0.05 ± 0.007 ^b^	0.60 ± 0.03 ^b^	0.062 ± 0.005 ^a^	1.31 ± 0.07 ^b^
		DS genotype						
Water	(−BS)	5.1 ± 0.3 ^ab^	5.0 ± 0.2 ^ab^	0.49 ± 0.01 ^b^	0.055 ± 0.005 ^b^	0.50 ± 0.03 ^b^	0.06 ± 0.007 ^b^	1.14 ± 0.03 ^ab^
	(+BS)	5.4 ± 0.4 ^a^	5.2 ± 0.2 ^a^	0.57 ± 0.06 ^a^	0.062 ± 0.006 ^a^	0.58 ± 0.04 ^a^	0.07 ± 0.009 ^a^	1.19 ± 0.04 ^a^
PEG	(−BS)	3.0 ± 0.2 ^bc^	3.8 ± 0.3 ^b^	0.34 ± 0.03 ^c^	0.031 ± 0.002 ^d^	0.35 ± 0.02 ^d^	0.037 ± 0.005 ^c^	0.81 ± 0.02 ^c^
	(+BS)	3.3 ± 0.3 ^b^	3.9 ± 0.2 ^b^	0.34 ± 0.02 ^c^	0.036 ± 0.005 ^c^	0.37 ± 0.03 ^c^	0.036 ± 0.008 ^c^	1.09 ± 0.05 ^b^

**Table 2:** Qualitative analysis of lignin accumulation in the basal part of the roots of wheat seedlings primed and unprimed with endophyte *B. subtilis* 10-4 (BS) after a 24 h exposure of 4-day-old seedlings on the solution of 12% polyethylene glycol-6000 (PEG) (*n* = 3). Water—non-stressed seedlings grown under normal conditions.

	Water		PEG	
	(−BS)	(+BS)	(−BS)	(+BS)
DT genotype	+	+++	+++	++++
DS genotype	+/−	++	++/−	+++

“−”—no staining; “+”—weak staining; “++”—intermediate staining; “+++”—strong staining; “++++”—very strong staining.

## Data Availability

Data are contained within the article.
